# Pelvic Actinomycosis Related to Intrauterine Device: A Case Report

**DOI:** 10.7759/cureus.80839

**Published:** 2025-03-19

**Authors:** Mariana Pereira, Susana Peres, Kamal Mansinho

**Affiliations:** 1 Infectious Diseases, Unidade Local de Saúde Lisboa Ocidental - Hospital Egas Moniz, Lisbon, PRT; 2 Infectious Diseases, Centro Hospitalar de Lisboa Ocidental, Lisbon, PRT

**Keywords:** actinomyces spp, chronic pelvic infection, intrauterine devices (iud), pelvic actinomycosis, uterine mass

## Abstract

Actinomycosis is a rare but persistent bacterial infection caused by *Actinomyces* spp., a Gram-positive bacillus that typically inhabits the oropharynx and gastrointestinal and urogenital tracts. It can infiltrate deeper tissues following trauma, surgery, or foreign body presence, forming granulomatous masses of filamentous bacilli. While cervicofacial actinomycosis is the most prevalent form, pelvic actinomycosis has been strongly associated with prolonged intrauterine device (IUD) use, where chronic irritation of the endothelium facilitates bacterial invasion. Diagnosing actinomycosis can be challenging due to its slow progression and clinical resemblance to malignancies. This report presents a case of a 71-year-old woman with postmenopausal uterine bleeding, abdominal mass, anemia, and weight loss. Imaging identified a complex pelvic mass, and surgical biopsy confirmed pelvic actinomycosis. The patient was treated with high-dose intravenous penicillin followed by prolonged oral antibiotic therapy. Additionally, a degraded, retained IUD suspected as the infection source was removed. A follow-up MRI showed a significant reduction in abscess size after one year of treatment. An actinomycosis diagnosis is crucial for effective treatment planning, with beta-lactam antibiotics being the cornerstone of therapy. However, severe cases may necessitate surgical drainage or resection. Given its ability to mimic other gynecological conditions, early recognition and appropriate management of pelvic actinomycosis, including prompt IUD removal when indicated, are essential for optimal patient outcomes.

## Introduction

Actinomycosis is a rare, invasive, indolent chronic bacterial disease caused by *Actinomyces spp.*, a filamentous Gram-positive bacillus commensal to the oropharynx, gastrointestinal tract, and urogenital tract. To date, 25 *Actinomyces* species isolated from human tissues have been described, the first being *A. israelii* in 1896. The species lives on mucous surfaces, accessing deeper tissues during trauma, surgery, or the presence of foreign bodies, destroying the mucous barrier and forming masses consisting of branched filamentous bacilli [1]. The bacterium has been observed in various anatomical sites (e.g., musculoskeletal system, respiratory tract, urogenital tract, digestive tract, central nervous system, skin, and soft tissue structures) [2]. According to the body site, actinomycosis can be categorized as cervicofacial, thoracic, and abdominopelvic forms. The most common clinical form of the disease is cervicofacial actinomycosis, representing approximately 60% of all reported cases [2].

Diagnosis of actinomycosis can be challenging, especially at the early stages, due to the disease's slow progress and nonspecific nature. Due to the spreading of the mass through tissue planes, it can even resemble a malignant tumor. Typical features of actinomycosis include abscess formation with sinus tracts and purulent discharge. The presence of sulfur granules and hard macroscopic grains in pus is considered among the hallmarks of actinomycotic lesions [1]; however, they are not always present.

*Actinomyces* is known to colonize the gastrointestinal flora, and evidence also suggests that asymptomatic female genital colonization occurs, with reports of different strains of *Actinomyces* being found, including *A. meyeri, A. neuii, A. radingae, A. turicensis, *and* A. urogenitali* [3]. In a review of over 20,000 cervical smears, the incidence of *Actinomyces*-like organisms was 0.26%, and most (81%) of the women who showed *Actinomyces*-like organisms in their cervical smears were intrauterine device users [4]. Pelvic actinomycosis has been associated with the presence of *Actinomyces* on an intrauterine device (IUD) after prolonged use, as the device can irritate the endothelium, causing erosion and trauma and facilitating the invasion of bacteria [3]. The ability of *A. israelli *to form a biofilm over copper surfaces in a synthetic intrauterine medium was demonstrated in an in vitro study, with the bacterium attaching and growing colonies on porous biofilm structures on copper plates [5]. In another study, cervical smears of 1,108 IUD users were screened, with a prevalence of *Actinomyces*-positive cervical smears among IUD users at 14%; no association between positive smears and the duration of placement of the IUDs was found [6]. However, cytology is not the most specific test for *Actinomyces*. Culture is the gold standard for the identification of *Actinomyces*; however, it is not routinely useful due to constraints in the techniques required for the isolation and identification of the species related to the microaerophilic or strict anaerobic character of *Actinomyces* and the necessity to prolong cultures on appropriate media and atmosphere [2]. Among 130 *Actinomyces* isolates from samples associated with IUDs sent to a laboratory between 1983 and 1999 for identification to the species level, one-third were identified as *A. israelii,* with its prevalence being double those of *A. turicensis, A. naeslundii, A. odontolyticus,* and *A. gerencseriae*, which were the following most common species [7].

This case report describes a postmenopausal woman diagnosed with pelvic actinomycosis related to a retained IUD following an extensive diagnostic workup of an intrauterine pelvic mass.

## Case presentation

**Figure 1:**
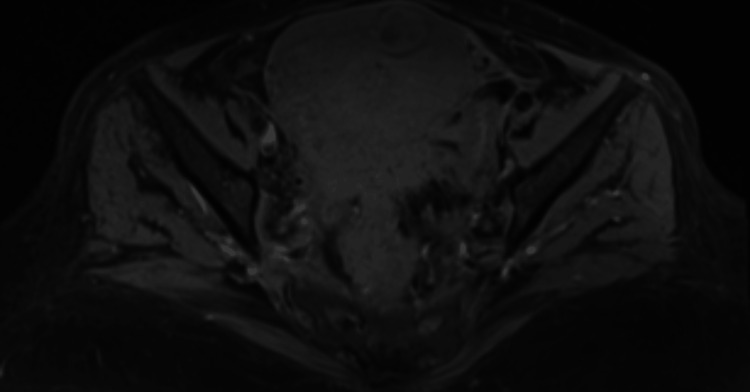
Pelvic MRI T1-weighted image showing an increased uterine volume, with nodular contours and an anteriorly located fibrotic nodule.

**Figure 2:**
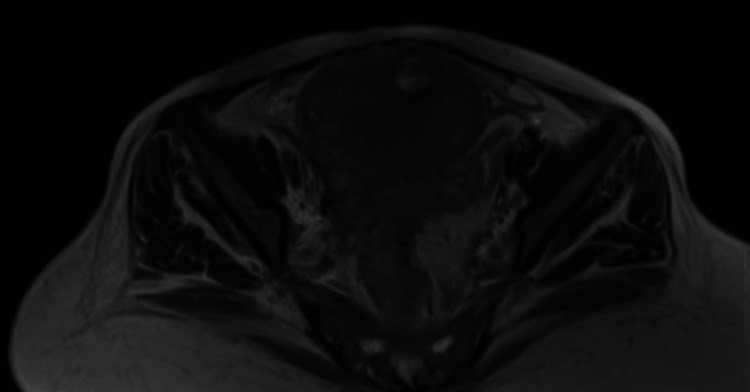
Pelvic MRI T2-weighted image showing the same hypointense fibrotic nodule in the anterior aspect of the uterine’s fundus with areas of cystic degeneration.

**Figure 3:**
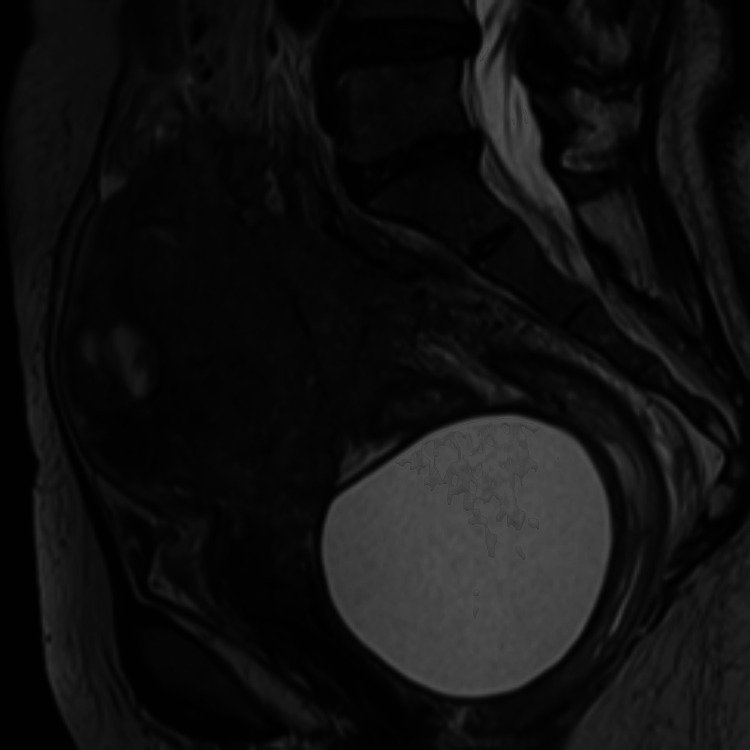
Pelvic MRI T2-weighted image revealing the presence of a large homogeneous, unilocular cystic formation located in the pre-rectal and retro-vaginal region, displacing anteriorly the cervix and vagina.

A 71-year-old woman with a medical history of hypertension, type 2 diabetes mellitus, dyslipidemia, hyperthyroidism, and obstetric history of G9P9A0 presents with a three-month history of postmenopausal uterine bleeding, hematochezia, anorexia, and a weight loss of 8 kg. At initial evaluation, the patient presented with a tender, 10 cm diameter, palpable lower quadrant abdominal mass. Blood work showed microcytic hypochromic anemia. The pelvic MRI showed: "Enlarged uterus (105 mm in longitudinal diameter) with irregular contours and displaying multiple nodular formations that are isointense in T1 and hypointense with areas of cystic degeneration in T2 (Figure 1). The largest nodule (83 mm in lateral-lateral diameter) is in the anterior region of the uterus, while another smaller nodule (17 mm in diameter in its greatest axis) in the anterior fundus appears fibrotic. These nodules occupy a significant portion of the myometrium and most likely indicate a diffuse fibromyomatous pattern (Figure 2). A large (82 mm), fluid-filled cyst is located behind the vagina and rectum, pushing the cervix and vagina forward (Figure 3). The right ovary is slightly enlarged and contains a 35 mm complex cyst with irregular contours and septations. Uterine enlargement and the cyst are likely causing right-sided kidney drainage issues (ureterohydronephrosis). Some intestinal thickening with inflammation and mild pelvic ascites is also noted."

The patient was referred to a general surgery consult and later was submitted to an exploratory laparoscopy and double J-stent placement. Intraoperatively, there was evidence of a bulky pelvic mass invading the sigmoid and ileal loops, and the mass was found to be unresectable, only a biopsy being performed. The histopathology report stated: “Fragments of abscess, mostly composed of histiocytes and neutrophils, along with some plasma cells and lymphocytes, are associated with well-defined basophilic nodular structures. These structures consist of filamentous bacteria with a radial growth pattern, sometimes forming a palisade at the periphery. The bacteria are Gram-positive and are also visible with Grocott staining. This sample has no evidence of dysplastic or malignant neoplastic tissue. Diagnosis: The histological and histochemical findings are consistent with pelvic actinomycosis.”

**Figure 4:**
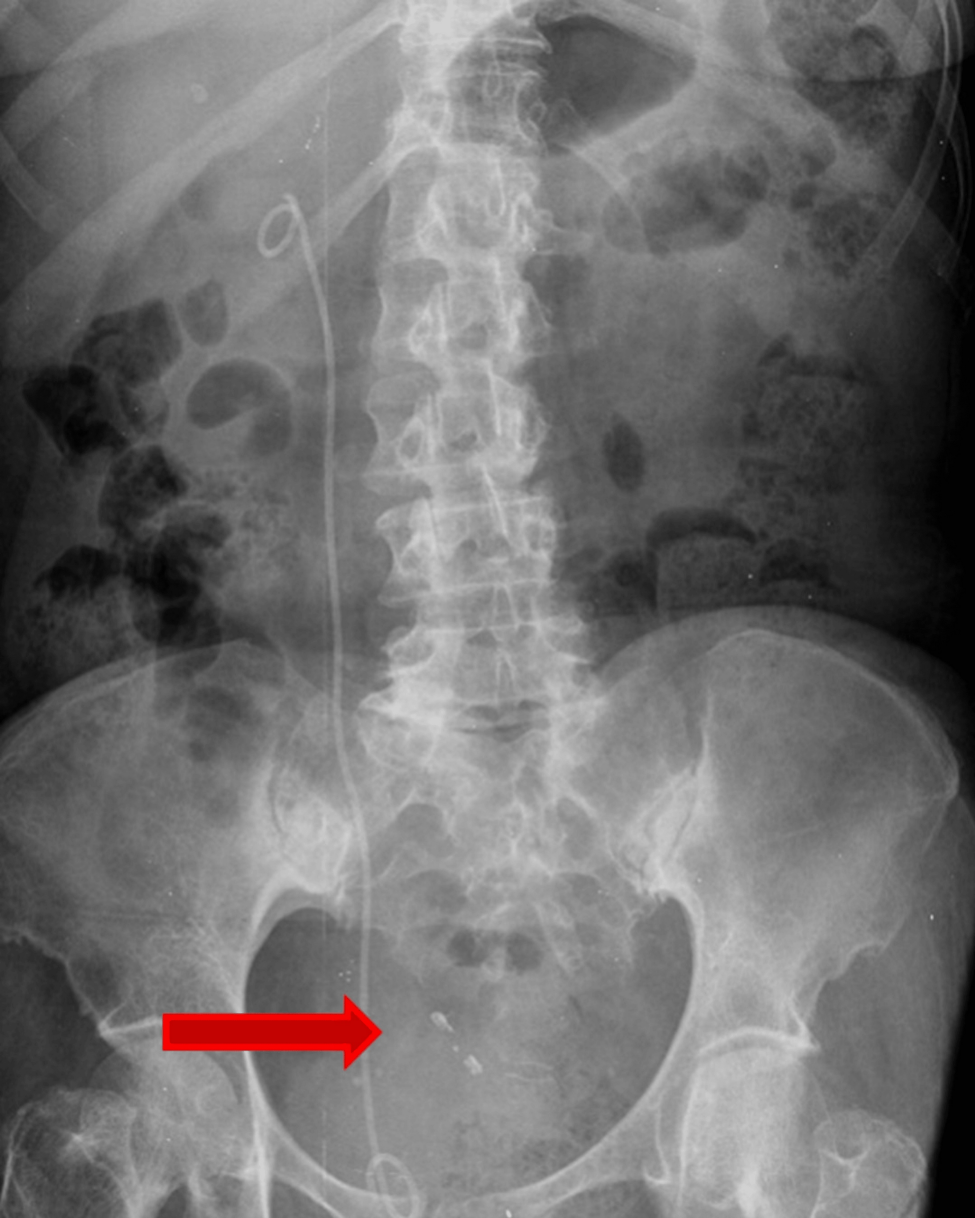
Abdominal X-Ray showing the presence of an opaque foreign body, compatible with an intrauterine device (arrow).

The case was discussed with the Infectious Diseases Department, and the patient began treatment with benzylpenicillin 24 MUI/day for 40 days, followed by a switch to oral amoxicillin 2 g twice daily (bid). The patient had an obstetric index of 9009, and she could not recall if she had a prior history of placement of an intrauterine device. A pelvic X-ray was performed, which showed an opaque image suggestive of an IUD (Figure 4). An intravaginal ultrasound corroborated the findings, showing a linear 30 mm hyperechoic body suggestive of an IUD. The patient underwent hysteroscopy with removal of the IUD, which was found to be in a state of advanced degradation as it was lodged in the isthmus of the uterus. The endometrial cavity showed signs of chronic inflammation and fibrosis, and a sample was sent for biopsy. The biopsy showed: “Several fragments of endometrial mucosa, predominantly showing an atrophic pattern with scarce glandular components, sometimes exhibiting squamous and eosinophilic metaplasia. The stroma is dense and hypercellular, with no significant atypia, but it contains an abundant mixed and histiocytic inflammatory infiltrate, along with extensive areas of fibrosis. Rare filamentous structures with a radial and basophilic growth pattern are also observed, consistent with *Actinomyces*. There is no evidence of hyperplasia, dysplasia, or malignant neoplastic tissue in this sample.”

**Figure 5:**
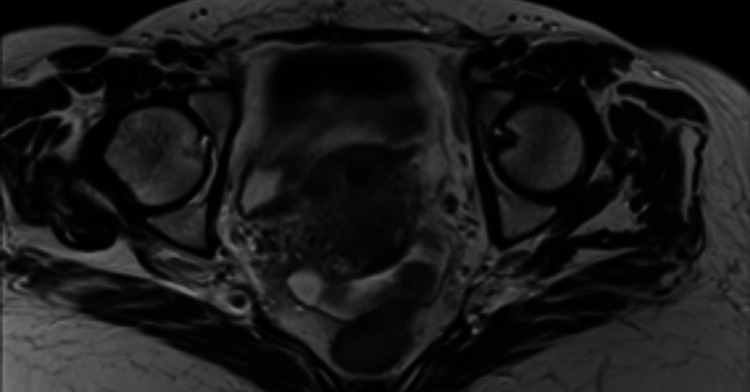
Pelvic MRI T1-weighted image at six months of antibiotic therapy showing a favorable evolution of the myometrial heterogeneity, with dimensional reduction of the hypointense areas, more evident in the posterior wall of the uterine body, without evidence of a cystic component.

**Figure 6:**
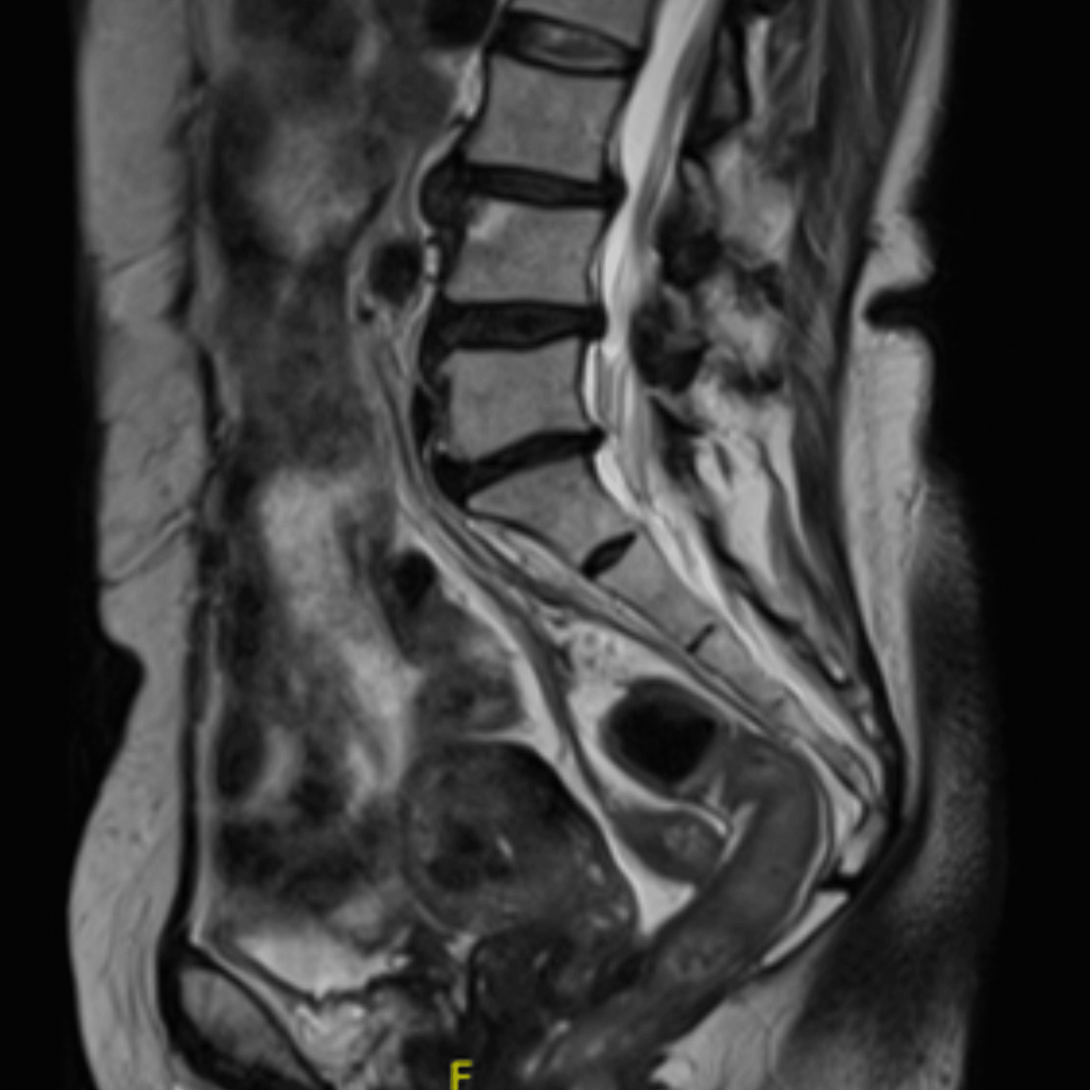
Pelvic MRI T2-weighted image at six months of antibiotic therapy revealing complete resolution of the collection in the Douglas pouch, which is now manifested by adhesions in the upper part of the rectovaginal septum.

The patient was discharged while maintaining treatment with oral amoxicillin 2 g bid. A follow-up pelvic MRI was performed at six months of antibiotic treatment, showing, compared to the previous MRI study: “A significant reduction in the size of the abscesses; however, some pelvic abscess masses persist, particularly affecting the posterior aspect of the uterine body, along with evident inflammatory changes in the fat surrounding the sigmoid mesocolon, mesorectum, and parametrial regions. A collection behind the uterus has also significantly reduced in size (Figures 5, 6)."

The patient completed one year of treatment with oral amoxicillin 2g bid with no significant adverse effects. A final pelvic MRI was performed after completion of treatment: “The uterus and cervix appear normal, with no abnormalities or lesions. The ovaries are atrophic, which is expected for the patient’s age, and show no tumors. There are some adhesions between intestinal loops in the pelvic area but no significant obstruction. A slight thickening of the peritoneal lining and a small amount of free fluid are present, but no concerning collections are seen. Mild fibrotic changes in the lower abdominal wall are noted, likely from past conditions. No ovarian masses are detected."

## Discussion

Diagnosing pelvic actinomycosis has several limitations. Symptoms are often nonspecific and slow to develop, making early detection difficult. Pelvic actinomycosis can mimic other gynecological diseases when presenting as a genital mass without fever, including malignant tumors, myomas, or adenomyosis. The infection can also spread through tissues, reaching the uterine tubes and causing salpingitis and abscesses, with subsequent destruction of the ovaries. The spread of the disease from the pelvic region to other organs such as the bladder, rectosigmoid colon, small bowel, and urethra has also been reported [8]. The most common symptoms of pelvic actinomycosis are lower abdominal pain, constipation, and vaginal discharge; fever can be present if a complication arises, such as peritonitis [2]. Imaging findings can also resemble other diseases, complicating differentiation. Microbiological diagnosis is challenging because *Actinomyces *species have slow growth and require prolonged culture periods. Histopathology is often needed for confirmation, but obtaining tissue samples is not always possible.

Women carrying an IUD for more than five years and presenting with a pelvic mass should have a high clinical suspicion of IUD-related actinomycosis. When suspecting IUD-related pelvic actinomycosis, the surgical removal of the IUD is crucial to acquiring clinical samples for microbiology and pathology analysis. IUD-related actinomycosis must be distinguished from IUD colonization, as no antimicrobial treatment is required for asymptomatic women who systematically change their IUD in the five years following insertion [2]. Additionally, actinomycosis can overlap with other IUD-related infections, and there is no routine screening for it, leading to potential delays in diagnosis.

Correctly diagnosing actinomycosis is fundamental for selecting the most suitable treatment plan and achieving favorable outcomes. Open surgical biopsy is often required for the definite diagnosis of pelvic actinomycosis; however, the prevailing trend is to minimize invasive procedures and instead focus on antibiotic regimens. Typically, abscesses need drainage for proper treatment, whereas total resection surgery may only be considered when there are extensive necrotic lesions or when antimicrobial therapy proves ineffective [1].

Regarding antibiotic treatment, overall, *Actinomyces* species are susceptible to beta-lactam antibiotics, such as penicillin, and to most treatments that target Gram-positive anaerobic rods, with the notable exception that the species is inherently resistant to metronidazole [1]. Patients typically undergo intravenous high-dose beta-lactam treatment for several weeks, followed by oral therapy lasting several months until up to a year. However, comprehensive data on the duration of antimicrobial treatment for such cases is lacking [2]. A retrospective review of 23 cases of abdominopelvic actinomycosis treated between 1994 and 2010, in which all patients underwent surgical intervention according to the mass's location and extent and received postoperative antibiotic therapy for durations ranging from less than two months to six months, showed that a combined surgical and antibiotic treatment resulted in a 91% improvement rate, with no recurrences reported over a median follow-up of 30 months, suggesting that a combination of surgical resection and prolonged antibiotic therapy is effective in treating abdominopelvic actinomycosis, yielding high success rates and minimal recurrence [9].

## Conclusions

Pelvic actinomycosis remains a rare but potentially significant complication associated with prolonged IUD use, often mimicking other gynecological conditions, including malignancy. Early recognition and diagnosis remain a challenge, highlighting the importance of a high index of suspicion in patients presenting with pelvic masses and a history of IUD use, followed by a thorough diagnostic workup guided by imaging and histopathological findings. To successfully manage this condition, a prolonged course of antibiotic therapy is often necessary, combined with surgical intervention when indicated. This case further emphasizes the importance of maintaining regular gynecological follow-ups and the potential risks of long-term IUD retention, particularly in postmenopausal women.

Due to pelvic actinomycosis's indolent nature, clinicians should remain aware of this condition when differentiating it from malignancies and other inflammatory conditions. Future research should explore strategies for early detection, optimal antibiotic regimens, indications for a surgical approach, and preventive measures, including patient education on IUD monitoring and timely removal.
